# Provider Implicit Bias in Prescribing HIV Pre-exposure Prophylaxis (PrEP) to People Who Inject Drugs

**DOI:** 10.1007/s11606-023-08040-7

**Published:** 2023-03-24

**Authors:** Alex Dubov, Douglas S. Krakower, Nicholas Rockwood, Susanne Montgomery, Steven Shoptaw

**Affiliations:** 1 School of Behavioral Health, Loma Linda University, Loma Linda, CA USA; 2 Division of Infectious Diseases, Department of Population Medicine, Beth Israel Deaconess Medical Center, Harvard Medical School, Boston, MA USA; 3 Department of Family Medicine, University of California Los Angeles, Los Angeles, CA USA

**Keywords:** clinical decision-making, pre-exposure prophylaxis, primary care, people who inject drugs, implicit bias

## Abstract

**Background:**

Multiple HIV outbreaks among people who inject drugs (PWIDs) have occurred in the USA since 2015, highlighting the need for additional HIV prevention tools. Despite high levels of need, pre-exposure prophylaxis (PrEP) is drastically underutilized among PWIDs. Implicit bias toward PWID held by clinicians may impede PrEP scale-up among these underserved patients. This study examined how primary care providers’ (PCPs) clinical decisions related to PrEP can be impacted by biases when the patient has a history of substance use.

**Methods:**

We conducted an online survey of PCPs (*n* = 208). The survey included the implicit association test (IAT) to assess unconscious attitudes toward PWIDs, direct questions regarding clinicians’ explicit PWID attitudes, and an embedded experiment in which we systematically varied the risk behavior of a hypothetical patient and asked PCPs to make clinical judgments.

**Results:**

A minority (32%) of PCPs reported explicit PWID bias. The IAT indicated strong implicit PWID bias (meant IAT score = 0.59, *p* < .0001) among 88% of the sample. Only 9% of PCPs had no implicit or explicit PWID bias. PWID patients were judged as less likely to adhere to a PrEP regimen, less responsible, and less HIV safety conscious than heterosexual or gay male patients. Anticipated lack of adherence mediated PCPs’ intent to prescribe PrEP to PWID.

**Conclusions:**

PCPs’ bias may contribute to PrEP being under-prescribed to PWID. Implicit and explicit PWID biases were common in our sample. This study illustrates the need to develop and test tailored interventions to decrease biases against PWID in primary care settings.

## INTRODUCTION

Over 15 million people inject drugs worldwide, of whom an estimated 3.2 million live with HIV.^1^ People who inject drugs (PWIDs) account for a disproportionate share of HIV prevalence and 10% of all new HIV diagnoses in the USA.^2^ Recent HIV outbreaks among rural communities of Indiana^3^, West Virginia^4^, and Arizona^5^, coupled with a dramatic increase in injection drug use in rural areas across the country^6^, underscores the need for effective HIV prevention services among PWID. This need is pronounced in 48 Ending the HIV Epidemic (EHE) priority counties that account for over half of all new HIV diagnoses in the USA.^7^ Among these are Riverside and San Bernardino counties in Southern California, collectively called the “Inland Empire (IE).” The IE, dubbed the “methamphetamine capital of the US,” is the transit point for over 25% of the US methamphetamine supply.^8^ This region is severely impacted by methamphetamine abuse which is also the main driver of new HIV cases.

Daily oral PrEP is recommended by the US Centers for Disease Control and Prevention (CDC) for HIV prevention among PWID,^9^ reducing HIV risk in this population by at least 74%.^10^ PrEP is often the only option for preventing HIV among PWID living in rural areas without access to other prevention efforts, such as syringe service programs (SSPs). PrEP is the only form of prevention that provides dual protection against both sexual and injection risks of acquiring HIV. According to the CDC estimates, 72,510 PWIDs have clinical indications for PrEP.^11^ The scale-up of PrEP among PWID is mainly absent, despite many PWIDs being likely to benefit and expressing a strong willingness to use PrEP.^12^ The underutilization of PrEP can be partially explained by clinicians who work with PWIDs and serve as gatekeepers due to implicit or explicit anti-PWID biases.^13^

We define implicit bias as negative attitudes or stereotypes that affect clinicians’ interactions, decisions, and actions toward PWID in an unconscious manner.^14^ Explicit bias, in turn, refers to conscious negative attitudes, often represented by discrimination and prejudice against PWID.^15^ Calabrese et al. have shown that disclosing injection drug use by a patient seeking PrEP may negatively influence providers’ clinical judgment and intent to prescribe PrEP.^16^ Therefore, clinicians’ prescribing decisions may be affected by implicit bias toward PWID seeking PrEP, though there is currently limited data to support this hypothesis.

This study aimed to measure implicit and explicit biases against PWIDs among a convenience sample of PCPs practicing in rural areas of IE. Additionally, we explored providers’ clinical decision-making around PrEP, including their assessment of PWID patients’ HIV risk without PrEP, candidacy for PrEP, and expectations for patients’ behavior with PrEP use (e.g., risk compensation). We assessed PrEP-related decision-making by presenting a chart of a hypothetical patient seeking PrEP and asking participants to make a series of clinical judgments about the patient. The patients’ HIV risk factors (injection drug use, sex with men, or sex with women) were systematically varied. We hypothesized that clinical assessments would vary according to patients’ HIV risk factors and mediate clinicians’ willingness to prescribe PrEP.

## METHODS

### Sample and Recruitment Strategy

Convenience sampling was used to recruit PCPs practicing in high HIV and substance use disorder (SUD) prevalence settings of IE. To be eligible, individuals needed to be English-speaking primary care providers licensed to prescribe PrEP. We defined primary care providers as Family Medicine or Internal Medicine physicians, nurse practitioners (NP), or physician assistants (PA) who provide care to adults in primary care clinics. We offered a $40 virtual Amazon gift card as a participation incentive.

### Data Collection Process

The data was collected from August 2020 until January 2021. The email invitation contained the eligibility criteria, confidentiality measures, incentive details, contact information for the primary investigator, and a unique link to the Qualtrics survey. After reviewing the study information and confirming their eligibility, physicians were directed to the study consent form (“click to consent” procedure) before completing the study survey. The survey took, on average, 20 min to complete. After completing the survey, participants were redirected to a separate page, where they could enter their email addresses to receive an Amazon virtual gift card. The Loma Linda University IRB approved this study.

### Measures

We collected clinicians’ demographic and professional characteristics. Implicit bias against PWID was measured using IATgen — a survey-based implicit association test (IAT) method described in detail in the methodological paper by Carpenter et al.^17^ Using this tool, we built a Qualtrics survey that contained a counterbalanced seven-block IAT. In each block, participants saw a stimulus (e.g., a word or image) on the screen. Stimuli represented “targets” (e.g., PWIDs or non-PWIDs) or the category (e.g., help-punishment). We used images (e.g., pictures of individuals injecting drugs or reading a book) as targets for PWIDs and non-PWIDs, respectively. We used words for Help (e.g., care, treatment) vs. Punishment (e.g., penalty, criminal) category exemplars. When stimuli appeared, the participant sorted the stimuli as rapidly as possible. We measured the response speed in milliseconds. Bias was inferred from response latencies.

Physician explicit bias against PWID was assessed using the drug use stigmatization scale (*α* = 0.88).^18^ This seven-item measure consists of negative statements about PWID, such as “substance users are weak-minded.” Responses to these 5-point Likert items ranged from (1) “Strongly Disagree” through (5) “Strongly Agree.” To explore the association of physicians’ attitudes about PWID with PrEP recommendations, we used three mock-up charts designed by Calabrese et al.^19^ and describing three different HIV risk factors: (1) heterosexual male patient who uses condoms inconsistently with one female sex partner who is HIV + and not in treatment; (2) MSM patient who uses condoms inconsistently with one male sex partner who is HIV + and not in treatment; (3) PWID with no current sex partners who inject heroin daily and reports occasional sharing of needles with HIV + injecting partner. This was a between-subject design, and each participant was randomized to review one chart. With respect to the patient described in the charts, we assessed clinicians’ estimates of the patient’s HIV acquisition risk both with and without PrEP; perception of the importance of the patient’s request to receive a PrEP prescription; hypothetical investment in helping the patient; judgments of the patient’s likelihood of adhering to PrEP, responsibility, safety-consciousness, and deservingness of help; and intent to prescribe PrEP.

### Analysis

#### Explicit Bias

Composite scores were created by summing the scores from the seven items (rated on a 5-point Likert scale). Higher scores (18–35) indicate more significant explicit stigma***.***

#### Implicit Bias

The IAT scoring procedures followed the recommendations by Greenwald et al.^20^ The IAT scoring algorithms create a D score, the difference between response latencies for the two critical category pairing conditions, divided by the standard deviations across all blocks. Scores between 0.15 and − 0.15 represent no preference for the non-PWID vs. the PWID (implying no bias against the PWID). Scores of 0.16 to 0.35 and 0.36 to 0.65 map to a slight and moderate preference for the non-PWID, respectively, and values greater than 0.65 show a strong preference for the non-PWID (suggesting strong bias against PWID).

#### Regression Model

We performed hierarchical linear regression analysis to predict explicit and implicit biases using the sample’s sociodemographic and medical training characteristics.

#### Between-Group Comparisons

Between-group comparisons were conducted using tests of differences (ANOVA and ordinal regression) to assess between-group differences in clinical judgments.

#### Correlation Analysis

We analyzed bivariate relationships separately for MSM-patient and PWID-patient conditions using Pearson correlation, Spearman rho, and phi coefficients.

#### Mediation Analysis

We used the Hayes’ PROCESS macro^21^ to conduct mediation analysis. This macro is designed for continuous or binary observed variables and has been commonly used in the social sciences to estimate indirect effects.^22^ PROCESS allows multiple mediators and provides bootstrapping as an estimation approach for statistical inference. As bootstrapping yields robust standard errors for indirect effects, we use this approach to obtain the estimates and present the results accordingly.

## RESULTS

### Demographics

Among 242 clinicians who accessed the survey and completed the consent form, 220 (90.9%) proceeded to the survey, and 208 (85.9%) completed the survey. The mean survey completion time was 20 min. Table 1 presents the descriptive characteristics of the sample. Physicians represented the majority (80.2%) of our sample, followed by nurse practitioners (12%) and physician assistants (7.8%). The average age of participants was 34.49 years (SD, 4.24), and the mean number of years in medical practice was 6.53 (SD, 3.53), with most respondents practicing in federally qualified centers (FQHCs). Slightly more than half (52%) were women, and 50% reported their race as white.

**Table 1:** Demographic Characteristics (*N* = 208)

Characteristics	*n* (%)
Age, mean (SD)	34.49 (4.24)
Gender	
Female	110 (52.8)
Male	98 (47.2)
Race	
Asian	81 (38.95)
Black/African American	20 (9.61)
Native American	3 (1.44)
White	104 (50.0)
Ethnicity	
Hispanic or Latino	22 (10.57)
Immigration status	
Foreign-born	64 (30.77)
Sexual orientation	
Homosexual (lesbian/gay)	21 (10.09)
Bisexual	10 (4.81)
Heterosexual	177 (85.1)
Clinical role and practice	
Physician	167 (80.28)
Nurse practitioner	25 (12.03)
Physician assistant	16 (7.69)
Number of years in clinical practice, mean (SD)	6.53 (3.53)
Number of SUD or OUD patients in care, mean (SD)	119.84 (70.7)

### Explicit PWID Bias

While most participants did not indicate responses consistent with a higher PWID-related stigma, one-third of participants did. For instance, approximately 45% of the sample thought that substance use is a moral failure, 22% acknowledged that PWID patients make them angry, and 20% answered “Agree” with the statement “PWID patients are dishonest.”

### Implicit PWID Bias

Clinicians’ IAT scores ranged from − 0.66 to 1.44, with a mean of 0.59 (SD = 0.41). This corresponds to a large effect (Cohen’s *d* = 1.42, *p* = 0.0001) and indicates a strong implicit bias against PWID among PCPs. Internal validity measures and error rates generated by the IATgen platform confirm these results as valid. There was significant divergence in the clinicians’ implicit and explicit biases against PWID (Table 2). Of the study participants, 67.8% reported a lack of explicit bias against PWID. However, 88% had an implicit bias against PWID, confirmed by their IAT scores. Even among respondents who did not express explicit bias, over half (58.17%) showed implicit bias.

**Table 2:** Prevalence of Explicit and Implicit Biases Among Primary Care Providers

	No implicit bias	Implicit bias	Total
No explicit bias	20 (9.61)	121 (58.17)	141 (67.78)
Explicit bias	5 (2.39)	62 (29.83)	67 (32.22)
Total	25 (12)	183 (88)	208

Explicit bias — values 18 + out of 35 possible

Implicit bias — values of 0.15 +

### Predicting Explicit and Implicit PWID Biases

Women and Black/African American respondents were less likely to report explicit bias against PWID patients (average PWID stigma scores 1.36 and 2.46 lower, Table 3). Compared to physicians and PAs, nurse practitioners reported significantly higher levels of explicit bias (average scores higher by 3.45 points). A higher number of SUD patients in one’s practice led to a modest reduction in explicit bias (− 0.005), while a higher number of years in clinical practice led to a slight increase in explicit bias (0.17). Age was a robust predictor of implicit bias (average IAT scores 0.58 points higher), and a quadratic effect of age was noted, such that younger and older clinicians exhibited higher levels of implicit bias. The magnitude of implicit bias was lower among those who did not report having received diversity training (average IAT scores 0.23 points lower).

**Table 3:**
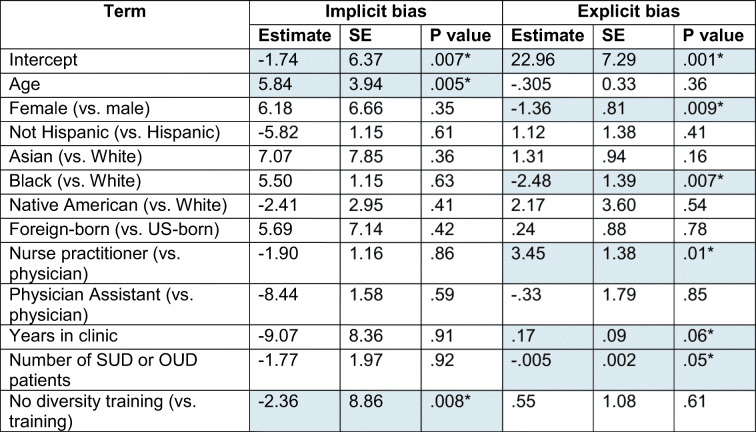
Predicting Explicit and Implicit Biases

Statistically significant values are highlighted in blue

### Between-Group Comparisons

Table 4 displays between-group mean comparisons, ANOVA, and ordinal regression results for the clinical judgments influencing PrEP prescribing decisions. Participants correctly judged both MSM and PWID patients as being at greater risk for HIV. Clinicians evaluated the risk of HIV while taking PrEP to be higher for PWID. Compared to other conditions, PWID patients were also judged as significantly less likely to adhere to a PrEP regimen, less responsible, and less safety conscious.

**Table 4:**
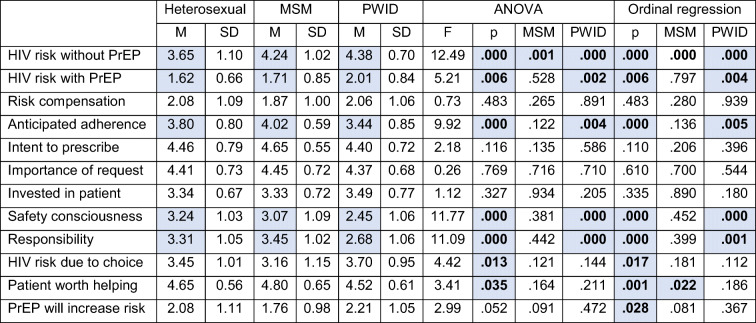
Between-Group Comparisons

Statistically significant values are highlighted in blue

### Correlation Analysis

Table 5 shows correlation coefficient values representing bivariate relationships among variables separately by conditions. In the *MSM condition*, clinicians with a higher number of patients living with HIV expected less risk compensation for their patients who take PrEP. Conversely, clinicians who were older and Asian thought their patients would remain at high risk of HIV while taking PrEP. Respondents who anticipated an increase in HIV risk among their patients on PrEP (risk compensation) were less likely to prescribe PrEP and deemed these patients less worthy of help. In the *PWID condition*, male (vs. female) clinicians judged their PWID patients as less responsible and deserving help. Clinicians with more SUD or patients living with HIV expected less risk compensation from their PWID patients on PrEP. Clinicians who anticipated risk compensation among their patients taking PrEP or less adherence to the PrEP regimen also considered these patients less responsible and worthy of help. The intention to prescribe PrEP to PWID patients was negatively associated with the anticipated likelihood of risk compensation. Clinicians were more likely to prescribe PrEP to the patients they considered responsible and worthy of help. These two categories were correlated with anticipated adherence to PrEP.

**Table 5:**
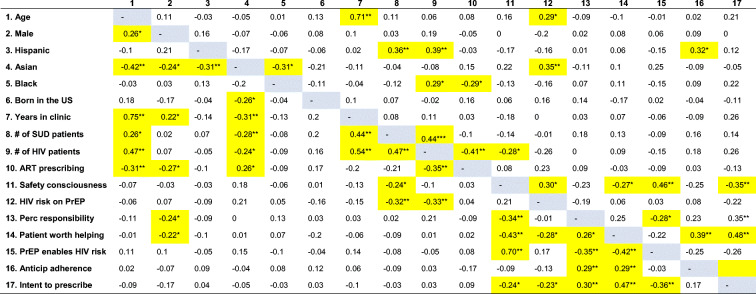
Correlation Analysis

Correlations for PWID condition below diagonal; correlations for MSM condition above diagonal. *p < 0.05; **p < 0.01. Statistically significant areas are highlighted in yellow

### Mediation Analysis

**Figure 1:**
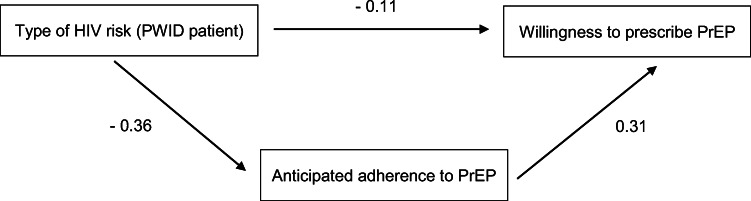
Mediation analysis.

Path coefficients are presented in Figure 1***.*** The mediation analysis examined the hypothesized role of anticipated adherence to PrEP as a mediator between the patient’s HIV risk factor (injection drug use vs. MSM with sexual exposure) and intention to prescribe PrEP. The mediating effect of anticipated adherence (indirect effect) was significant according to bootstrapping results (*b* =  − 0.114 [− 10.212, − 0.03]), as the confidence intervals did not include zero. PWID patients were judged as less likely to adhere to PrEP (*b* =  − 0.364 [− 0.611, − 0.117]) which was in turn independently and significantly associated with lower willingness to prescribe PrEP (*b* = 0.313 [0.195, 0.432]).

## DISCUSSION

In our study, a hypothetical PWID patient seeking PrEP in a primary care clinic had less than a 10% chance of encountering an unbiased clinician. These findings point to the clinicians’ bias as a potential factor contributing to the under-prescription of PrEP to PWID. Several recent studies^23,24^ suggest that PWIDs are prescribed PrEP less often than other priority populations. For instance, 76% of physicians in one study had no PWID patients on PrEP,^25^ while data from San Francisco^26^ and Boston^27^ indicate that only 2–3% of PWIDs were prescribed PrEP. Yet these studies had rarely examined the outsized role of clinicians’ bias in low PrEP prescription among PWIDs. Our study is among the first to provide empirical evidence that primary care providers’ clinical decisions related to PrEP may reflect explicit and implicit biases when the patient is PWID, compared to heterosexual or MSM patients who are similarly at risk for HIV.

The prevalence (88%) and the magnitude (mean *d* score = 0.59) of the implicit bias in our study highlight the need for clinicians to address the role of implicit biases in PrEP disparities among PWIDs. One in three clinicians also exhibited explicit bias, considering substance use as a moral failure and admitting to frustration with PWIDs as patients. These findings suggest that PWID bias is rooted in the belief that addiction is a personal choice reflecting a lack of willpower and a moral failing. Furthermore, the results demonstrate that explicit and implicit anti-PWID biases are poorly correlated. Over half (58.17%) of clinicians reported a lack of explicit bias yet had IAT scores indicating implicit bias. This discrepancy means that even well-intentioned clinicians may contribute to disparities in PrEP access through biases operating outside their conscious awareness.

Not all PCPs hold the same levels of implicit and explicit biases toward PWID. Consistent with the literature,^28^ nurse practitioners in our sample expressed significantly greater explicit bias. This finding suggests the need to understand why some professional groups report higher implicit bias than others. Women and Black/African American participants reported lower levels of explicit bias, while older clinicians reported higher implicit bias than younger clinicians. When examining levels of implicit bias related to other patients’ attributes (e.g., race, weight), studies found similar trends of lower bias among women^29,30^ and Black/African American clinicians^31,32^ and higher bias among older providers.^33,34^ Whereas one might expect clinical expertise and a history of diversity training to mitigate bias levels, our data did not support this assumption. Instead, clinical experience was associated with slightly higher levels of explicit bias, suggesting that if the balance of the contact between clinicians and patients who use substances includes challenging or negative experiences, it may promote the development of negative implicit associations toward this group of patients. Diversity training was associated with greater implicit PWID bias, pointing to the need of developing targeted stigma-reduction interventions.

Attribution theory suggests that when considering the causes of social problems, people typically exaggerate the role of individual responsibility and underestimate the importance of factors outside of individuals’ control.^35^ Stigma research demonstrated that this attribution bias, such as high perceived controllability over a disease, contributes to increased bias among clinicians.^36^ Similarly, in our study, clinicians considered PWID patients’ HIV risk a function of controllable choices. This clinical assessment correlated with providers being less inclined to perceive PWID patients as deserving help. Attributing personal responsibility for substance use or HIV risk to the people who experience substance use disorder correlates with an increased degree of clinicians’ bias and a lower likelihood that these patients will receive the help they need from their clinicians to protect themselves from HIV.

Our comparison of clinical decision-making across risk groups highlights several stereotypes to be targeted in interventions to address bias against PWID. The PWID patient was judged to be less responsible and less likely to adhere to PrEP. Clinicians also considered the PWID patient less HIV safety conscious, even though he was proactively seeking PrEP, which indicates his concern about HIV and his health. Additionally, both MSM and heterosexual patients reported condomless sex, while the PWID patient had no sexual partners. The assessments of personal concern about HIV safety were similar between MSM and heterosexual patients. Still, they were much lower for the PWID patient, suggesting harsher judgments attributed to substance use stemming from clinicians’ bias.

The findings of perceived lack of responsibility, expected poor adherence to PrEP, and lack of safety-consciousness attributed to PWID patients by PCPs need to be contrasted with the evolving evidence about PrEP interest and adherence among PWID. In a recent survey^37^ of over 300 MSM with substance use, adherence to PrEP determined by dried blood samples was 89% at week 12 and 83% at week 48. Similarly, multiple studies^38,39^ indicate that PWIDs are largely unaware of PrEP but demonstrate high interest in PrEP upon gaining awareness. The lack of PrEP awareness among PWID also points to the need for clinicians to initiate conversations about PrEP. Without effective training to improve clinicians’ willingness to prescribe PrEP for PWID, biases and stereotypes may preclude these conversations and exacerbate PrEP disparities.

In our study, many clinicians exhibited greater PrEP pessimism for PWID compared to other conditions, believing that PrEP would not help PWID solve their long-term HIV risk. This pessimism can be explained by clinicians prioritizing harm-reduction interventions, while forgetting about overlapping sexual and injection HIV risks and the fact that offering PrEP to PWID may increase their access to harm-reduction services. PrEP pessimism among providers points to the need to communicate evidence of the efficacy of PrEP among PWID patients as one of the strategies to reduce bias and increase prescribing.

Finally, anticipated adherence significantly mediated the association between the risk group (PWID vs. MSM) and clinicians’ willingness to prescribe PrEP. That is, PWIDs were judged to be less likely to adhere, which was associated with lower willingness to prescribe PrEP. These adherence concerns had no basis in the clinical scenario presented to the providers, in which the patient is an excellent PrEP candidate proactively seeking preventive care. Stereotypes about poor adherence to PrEP among PWID patients may coincide with the belief that prescribing PrEP to poorly adherent patients could be more harmful than helpful. Increasing clinicians’ awareness of the evidence that HIV drug resistance with PrEP use rarely occurs and that PrEP provides robust HIV protection even with periodic missing doses may alleviate adherence concerns.^40^ Furthermore, introducing the long-acting injectable PrEP may help overcome barriers to PrEP implementation among PWID.

An important limitation of this study is that the sample is not representative of PCPs everywhere. It is possible that selection bias led us to underestimate or overestimate the presence of implicit and explicit biases toward PWID patients among PCPs. We attempted to examine the impact of provider bias on PrEP-related medical decision-making using a mock medical chart. This approach may not accurately assess or activate the mechanism by which implicit bias influences the decision to prescribe PrEP. As disparities in HIV prevention and treatment are driven by differences in patient-provider communication, real-world or simulated studies may provide a more accurate assessment of PrEP-related decision-making. Implicit bias shows geographic variation,^41^ and this study was conducted in a rural and more conservative setting, resulting in potentially higher levels of bias. Finally, it is worth acknowledging concerns about whether results on the IAT truly predict behavior or whether the accepted IAT methodology is sound. Despite these concerns, a large body of research^42^ supports the reliability and validity of the IAT. Since we developed novel IAT for this study, additional studies should validate it.

This study provides empirical evidence of how PCPs’ clinical decisions related to PrEP may be affected by biases when the patient is PWID. Our findings highlight the necessity to design interventions teaching clinicians to identify and overcome biases in their clinical interactions with PWID. Research suggests^43^ that implicit bias can be changed with deliberate effort, at least in the short term. For instance, academic detailing^44^ is one of the evidence-based outreach education strategies successfully used to support the scale-up of PrEP and target HIV and substance use–related biases. Considering the high burden of substance use disorders in the USA, it is critical to develop and implement effective strategies to prevent PCPs’ bias from negatively affecting clinical outcomes or contributing to health disparities among PWID.
